# Understanding Limitations in Electrochemical Conversion to CO at Low CO_2_ Concentrations

**DOI:** 10.1021/acsenergylett.4c01224

**Published:** 2024-06-24

**Authors:** Danielle A. Henckel, Prantik Saha, Sunil Rajana, Carlos Baez-Cotto, Audrey K. Taylor, Zengcai Liu, Michael G. Resch, Richard I. Masel, K. C. Neyerlin

**Affiliations:** † National Renewable Energy Laboratory, 15013 Denver W Parkway, Golden, Colorado 80401, United States; ‡ Dioxide Materials, 1100 Holland Dr., Boca Raton, Florida 33487, United States

## Abstract

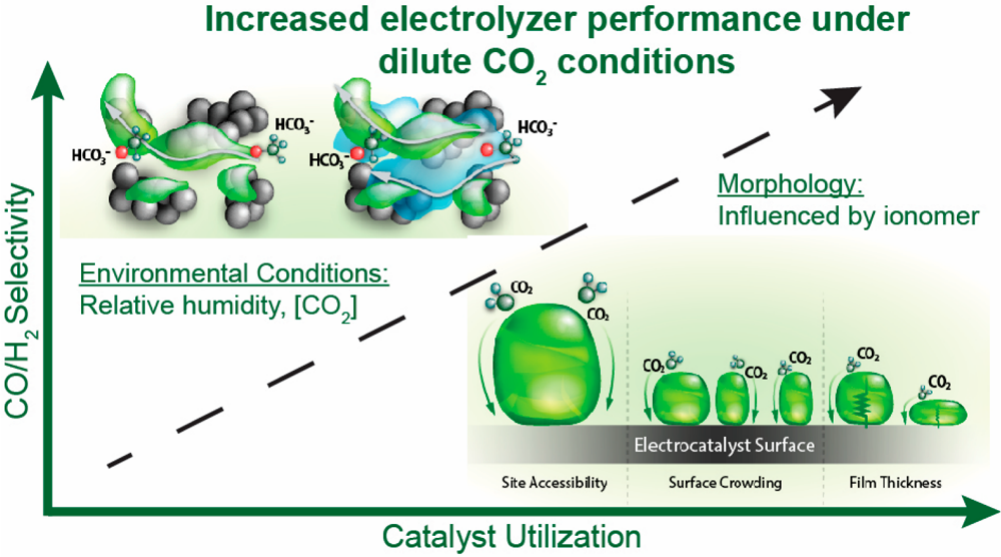

Low-temperature electrochemical CO_2_ reduction has demonstrated high selectivity for CO when devices are operated with pure CO_2_ streams. However, there is currently a dearth of knowledge for systems operating below 30% CO_2_, a regime interesting for coupling electrochemical devices with CO_2_ point sources. Here we examine the influence of ionomer chemistry and cell operating conditions on the CO selectivity at low CO_2_ concentrations. Utilizing advanced electrochemical diagnostics, values for cathode catalyst layer ionic resistance and electrocatalyst capacitance as a function of relative humidity (RH) were extracted and correlated with selectivity and catalyst utilization. Staying above 20% CO_2_ concentration with at least a 50% cathode RH resulted in >95% CO/H_2_ selectivity regardless of the ionomer chemistry. At 10% CO_2_, however, >95% CO/H_2_ selectivity was only obtained at 95% RH under scenarios where the resulting electrode morphology enabled high catalyst utilization.

## Introduction

The electrochemical conversion of CO_2_ into commodity chemicals is currently being investigated to utilize low-carbon electricity into the chemical industry and upgrade CO_2_ into value-added products. Of the available candidate chemicals to be produced electrochemically from CO_2_, CO has the largest market price of CO_2_ reduction products and technologies for the electrochemical conversion are increasingly mature.^1−4^ Since the largest amounts of CO_2_ emitted are from industrial sources at less than 30%,^5,6^ and the enrichment of CO_2_ streams from carbon capture will be cost-intensive, CO_2_ to CO at low concentrations of CO_2_ could greatly impact industrial decarbonization. To date, most work on CO_2_ to CO conversion has focused on pure feed streams of 100% CO_2_ and relatively few studies have investigated electrocatalytic activity at low concentrations of CO_2_.^7−9^

Currently, CO_2_ to CO electrolyzers can run at >95% CO/H_2_ selectivity at 200 mA/cm^2^ and ≤3 V in a zero gap configuration for greater than 3500 h.^10^ This high selectivity for CO_2_ to CO can be realized by a zero gap system utilizing the anion exchange ionomer polystyrene 1,2,3,4-tetramethylimidazolium chloride (Sustainion) and an anion exchange membrane incorporating the same imidazolium functionality. In this work, we extend this previous zero gap configuration to focus on CO/H_2_ selectivity at low concentrations of CO_2_. In order to tackle the decreasing CO selectivity with decreasing CO_2_ concentration, other work has focused on catalyst development, water crossover, CO_2_ pressure, electrochemical pulsing, and ionomer distribution.^8,9,11,12^ In this work, we focus instead on the impact of ionomer chemistry and its resulting influence on the electrode morphology and electrochemical properties to improve CO selectivity at low CO_2_ concentrations.

The surface electric field plays an important role in selectivity for the electrochemical reduction of CO_2_ and has been suggested to increase the local concentration of CO_2_ at the surface by stabilizing negatively charged intermediates and preventing diffusion of H_3_O^+^, thus synergistically promoting CO_2_ reduction over the hydrogen evolution reaction (HER).^13−17^ While most work has focused on the role that cations play on the surface electric field of the electrode, this can also be induced by cationic ionomer groups.^18^ Utilizing a higher concentration of electrolyte cations and cationic ionomers purportedly suppresses the HER and enables CO_2_ reduction at low pH. The same strategy could be effective when using low concentrations of CO_2_, where competition with HER is also a concern. In this study, we explore different cationic group ionomers (imidazolium and piperidinium) that vary in degree of localized charge and hydrophobicity to understand the impact these properties (water transport, stabilization of charged intermediates) have on CO_2_ to CO conversion at low CO_2_ concentrations. By identifying the underlying ionomer properties that lead to improved performance, we can design more efficient catalyst layers for this reaction.

### Characterization of Catalyst Layers

To examine the electrode morphology, we obtained scanning electron microscopy (SEM) images (Figures S2–S4). From these images, we can see similar catalyst agglomerates for the high-resolution images and a similar, smooth coating on the low-resolution images with the Ag catalyst completely covering the gas diffusion media. Additionally, we performed EDS on the electrodes to detect the Cl^–^ counterion associated with the ionomers. From Figures S5–S7, the Cl^–^ signal is evenly distributed, demonstrating that, qualitatively, all the ionomers have been well incorporated into the catalyst layers. Nominally a well-distributed ionomer network in the catalyst layer has been attributed to promotion of ion transport pathways.^12^ Previous work by Saha et al. identified that cathode ionomer and ions from anolyte crossover equally influence the ionic conductivity in CO_2_ cathodes.^19^ The function of the ionomer is likely 2-fold; one to facilitate ionic conduction and two to help disperse the catalyst in the ink and influence the resulting electrode morphology.

### CO/H_2_ Selectivity at Different Concentrations of CO_2_

**Figure 1:**
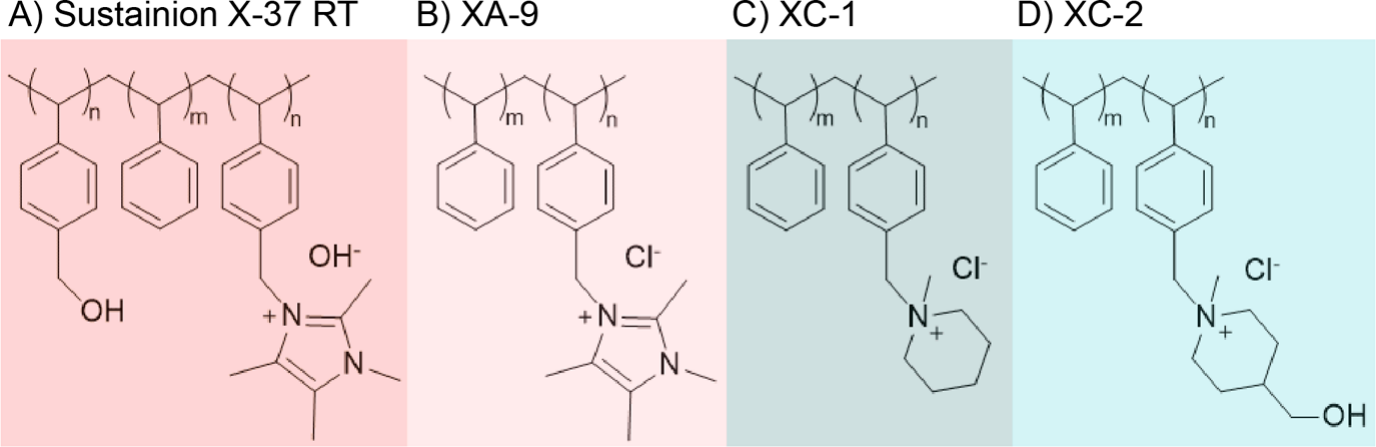
Structures of polymers used in this study: (A) Sustainion X-37 RT (membrane); (B) XA-9; (C) XC-1; (D) XC-2 (ionomer polymers).

**Figure 2:**
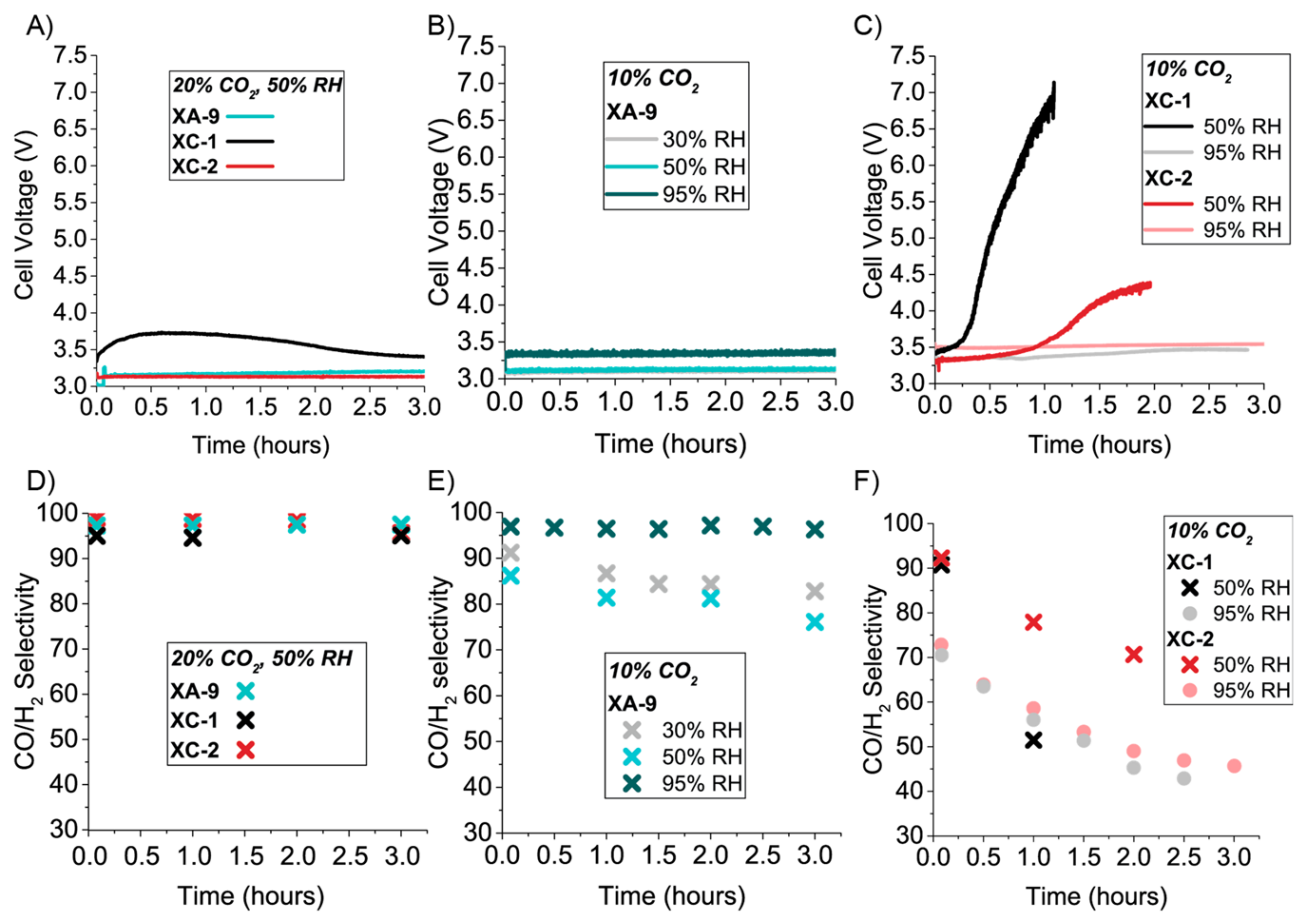
Time versus cell voltage for a 25 cm^2^ cell operating at 200 mA/cm^2^ for (A) XA-9, XC-1, and XC-2 at 20% CO_2_ and 50% RH, (B) XA-9 at 10% CO_2_ and 30, 50, and 95% RH, and (C) XC-1 and XC-2 at 10% CO_2_ and 50 and 95% RH. (D–F) Corresponding CO/H_2_ selectivities for data in (A–C).

As mentioned in the Introduction, current CO_2_ to CO electrolyzers can maintain >95% CO/H_2_ selectivity at 200 mA/cm^2^, when utilizing 100% CO_2_ and operating at 100% relative humidity (RH) feed.^10^ In order to understand the effect of the CO_2_ concentration and RH on the CO/H_2_ selectivity, we tested Ag cathodes utilizing three different ionomers (labeled as XA-9, XC-1, and XC-2, see Figure 1) across a range of different operating conditions. Figure 2A,D show the cell voltage and product selectivity for XA-9, XC-1, and XC-2 under conditions of 20% CO_2_ and 50% RH at 200 mA/cm^2^. All three ionomers maintain a CO/H_2_ selectivity of >95% for 3 h at these conditions. Both the XA-9 and XC-2 electrodes maintain a voltage below 3.3 V, with the XC-1 electrode at a voltage of ∼150 mV higher. While all three electrodes had similar performance at 20% CO_2_ and 50% RH, this was not the case when they were operated under more challenging conditions. Figure 2B,E shows the cell voltage and CO/H_2_ selectivity for the XA-9 electrode at 10% CO_2_ and 30, 50, and 95% RH. From the most challenging experiment of 10% CO_2_ with 30% RH, we found that the CO/H_2_ selectivity was <90% and continued to drop over 3 h. While increasing the RH to 50% did not improve the selectivity, increasing the RH to 95% allowed the selectivity to remain >95% for 3 h with a stable voltage of ∼3.4 V.

Similar to the XA-9 electrodes at low RH and 10% CO_2_, the XC-1 and XC-2 electrodes also had decreased performance, though the voltage increase was exacerbated. The voltage and CO/H_2_ selectivity can be seen in Figure 2C,F, respectively, for the XC-1- and XC-2-incorporated electrodes at 10% CO_2_ and 50% and 95% RH. As seen in Figure 2C, the voltage profiles for XC-1 and XC-2 increased over time at 10% CO_2_ and 50% RH. The cell voltage from the XC-1 electrode increased rapidly to 7 V within the first hour, and the voltage from the XC-2 electrode increased to 4.5 V at the 2 h mark. The CO/H_2_ selectivity for the XC-2 electrode decreased to <75% at 2 h and declined even faster for the XC-1 electrodes to ∼50% at 1 h. Notably, the voltage increase for both systems was much larger than expected from anticipated Nernstian voltage change due to a reduced CO_2_ concentration (∼9 mV). Suspecting that the increased voltage was the result of restricted ion transport pathways in the catalyst layer,^12^ the cell RH was increased to 95%. As seen in Figure 2C, this stabilized the cell voltages for all three electrodes, keeping them below 3.5 V for the duration of the test. However, CO/H_2_ selectivity still decreased for both XC-1 and XC-2 electrodes to <50% within 3 h.

From the data set in Figure 2, we uncover two interesting aspects of CO_2_ to CO conversion at low CO_2_ concentrations under our conditions. First, humidity is an important factor for maintaining a consistent low cell voltage, and second, variations in ionomer composition can impact CO selectivity. Since the selectivity decreases over several hours, and an increase in humidity can mitigate this loss of selectivity in the case of the XA-9 electrode, we hypothesized that the gradual decrease in CO/H_2_ selectivity might be due to a decrease in catalyst utilization.^20^

In order to understand the mechanism for increased voltages from XC-1 and XC-2, we performed a voltage recovery experiment (Figure S11). In this experiment (XC-1, 50% RH), we varied the CO_2_ concentration from 100% to 10% and the voltage change was instantaneous, increasing from ∼3 to 7 V within 10 min. This voltage change is reversible, and upon raising the CO_2_ feed to 100%, the voltage decreased back to the baseline. Due to the instantaneous nature of the voltage response, we consider that the high voltages for XC-1 and XC-2 may be due to a lower concentration of CO_2_ throughout the electrode. While the direct interface with the membrane should be wetted for the reaction to occur there, the reaction must move further into the catalyst layer to maintain the same rates under lower reactant conditions. If access to the entire catalyst layer is inhibited through low catalyst utilization, the cell voltage will increase and the selectivity will decrease. The increase in RH stabilized the voltage for XC-1 and XC-2, but still led to a decrease in CO/H_2_ selectivity. In this case, the hydrogen evolution reaction (HER) is increasingly preferred, likely due to an increase in the H_2_O/CO_2_ ratio.

### Relative Capacitance as a Function of RH

**Figure 3:**
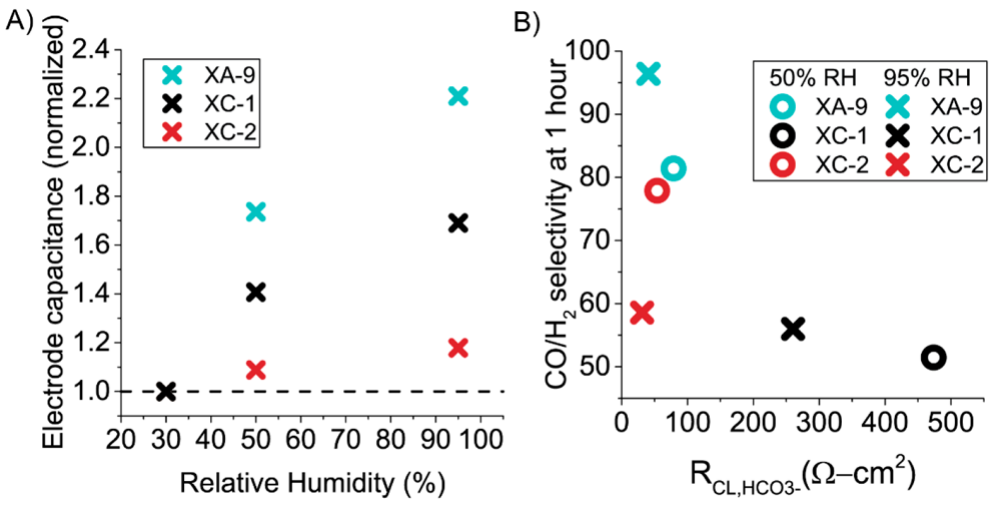
(A) Relative humidity (%) versus normalized electrode capacitance (all values are normalized to capacitance at 30% RH for respective electrodes). (B) Ionic resistance of the catalyst layer (*R*_CL,HCO_3_^–^_ (Ω cm^2^)) versus CO/H_2_ selectivity at 1 h at 200 mA/cm^2^ for XA-9, XC-1, and XC-2 at 10% CO_2_ and 50% and 95% RH.

Electrochemical impedance spectroscopy (EIS) was used to elucidate the maximum available electrochemical surface area as a function of RH. Catalyst utilization describes the fraction of catalyst that is available to the electrochemical reaction (for discussion, see refs (21 and 22)).These experiments were performed as previously described (Figure S12).^19^ Briefly, we will measure the EIS in a region where no or little charge transfer is occurring. This will allow us to fit our data to a transmission line model and extract the resistance to charge transfer within the catalyst layer.^19^ In this configuration, the anode (Pt/C with H_2_) serves two purposes: as a reference hydrogen electrode (RHE) and as a counter electrode performing either a hydrogen oxidation reaction (HOR) or hydrogen evolution reaction (HER), depending on the voltage bias. The cathode is still separated from the electrolyte with a membrane, so conditions such as electrolyte crossover are reflective of those during the CO_2_R experiments. We varied the RH of the cathode gas inlet and measured the EIS in a potential region where only the double-layer capacitance for Ag dominates (0.85 V vs RHE; this potential is near the open circuit potential of the electrode and is likely due to the presence of oxides on the surface of the Ag). From the EIS, we extracted the capacitance of the electrodes and normalized them to the capacitance at 30% RH (Figure 3A). Interestingly, all three electrodes had different water uptake properties, with XA-9’s capacitance increasing 2.2 times from 30% to 95% RH. In contrast, the XC-1 and XC-2 electrodes have a lower capacitance response to RH, increasing by 1.7 and 1.2 times, respectively. The increase in capacitance as a function of RH is a result of capillary condensation filling pores and providing increased ion conducting pathways. Capillary condensation occurs when the pore vapor pressure or capillary pressure is greater than the water saturation vapor pressure. The vapor pressure inside pores can be greater than the water saturation pressure due to the van der Waals interactions inside the pore. This effect allows water to condense more readily in pores with smaller pore sizes undergoing capillary condensation at lower RH. From the data in Figure 3, we hypothesize that the average capillary pressure is greater in the order XA-9 > XC-1 > XC-2. The average capillary pressure in this system can by influenced by pore size, pore volume, or the surface tension of the intruding liquid (influenced by the additives or surfactants).^20,23,24^

In these systems, water can be provided either by the anolyte (from membrane crossover) or by the cathode feed gas water vapor. Previously, it has been shown that, in Cu electrodes in a similar membrane electrode assembly, an increase in cathode gas RH increased the surface conductivity by 1.5 times from 0 to 100% RH, even with KOH electrolyte present.^19^ As seen in Figure 3, the electrode capacitance is influenced by the relative humidity of the gas stream, which demonstrates that the electrodes are not fully wetted by the electrolyte crossover alone. As seen in the performance data for XC-1 and XC-2, the voltage rapidly increases when at 50% RH but is stable at 95% RH. This could be due to a loss of electrode capacitance at lower RHs.

An interesting aspect of this work is that the ionomer appears to influence the water distribution in the catalyst layer. The capillary pressure of pores can be influenced by surfactants, so it is possible that the hydrophobicity of the ionomer can play a role in the difference in average capillary pressure for the electrodes.^23−25^ For example, this has been shown in the case of carbon electrodes, where Nafion content increased wetting.^26^ The increase in relative electrode capacitance with relative humidity from Figure 3A correlates with the expected hydrophobicity of the ionomers XA-9 > XC-1 > XC-2. The charge-delocalized imidazolium group of the XA-9 ionomer will be more hydrophilic than the piperidinium groups of XC-1 and XC-2.^27^ Additionally, the XC-1 ionomer contains a methylhydroxy group, increasing the ionomer’s hydrophilicity. While catalyst layer hydrophobicity^28^ and increased water in the catalyst layer^12,29^ are typically correlated with an increased CO_2_R over the HER, in this case an increase in feed gas humidity is increasing the electrode capacitance and therefore catalyst utilization. Additionally, other work with membrane electrode assemblies for CO_2_ CO conversion has noted the advantageous role of increased water in the catalyst layer. Kim et al. noted that increased water crossover, through flow rate, increased the FE for CO^9^ and work by Wei et al. has demonstrated that increased water in the catalyst layer, through ionomer incorporation, increases the formation of the *COOH intermediate, thereby increasing CO FE.^30^ Although other work has described amine-based additives as increasing the local CO_2_ concentration in the catalyst layer,^31,32^ we do not expect this to be a significant effect due to the lack of a lone pair on the nitrogen in the charged ionomers used in this work.

### Ion Transport through the Catalyst Layer

We can also extract the resistance to ion transport (*R*_CL,HCO_3_^–^_) through the catalyst layer from the EIS. A higher resistance to ion transport indicates that the ion transport through the catalyst is inhibited, potentially originating from disconnected ionomer pathways in the catalyst layer^33^ or ionomer type.^34^ If the catalyst layer has a higher ionic resistance, the effective catalyst surface area will be lower than in the geometric catalyst layer, resulting in an uneven current distribution throughout the electrode.^21^

Resistance to ion transport of HCO_3_^–^ (HCO_3_^–^ is assumed to the charge carrier in this specific measurement due to the low current conditions) versus the CO/H_2_ selectivity at 1 h can be seen in Figure 3B. The ionomer XC-1 has a significantly higher *R*_CL,HCO_3_^–^_ than the other ionomers by 2 orders of magnitude. This means that the catalyst utilization for XC-1 will be much lower than those for the other electrodes, which is reflected in its lower CO/H_2_ selectivity. Relative humidity also has an effect on *R*_CL,HCO_3_^–^_ increasing this value by ∼200 Ω cm^2^, from 95% to 50% RH for the XC-1 electrode due to increased access within the catalyst layer. The XC-2 electrode has a *R*_CL,HCO_3_^–^_ value comparable to that of the XA-9 electrode, <50 Ω cm^2^. However, its decreased ability to increase capacitance as a function of RH may lead to lower catalyst utilization and an increase in *R*_CL,HCO_3_^–^_ at longer times (increased voltage in Figure 2C at 2 h). The *R*_CL,HCO_3_^–^_ data do not correlate with the ion exchange capacity (IEC) data of the ionomer polymers alone. We measured the IEC of the XA-9, XC-1, and XC-2 ionomer polymers (Table S1), as this could affect the ability of the ionomer to transport charge. Interestingly the XC-1 and XC-2 ionomers have a similar (∼1.39 mM/g) ion exchange capacity (IEC), higher than that of XA-9 (0.94 mM/g). We hypothesize that other factors, such as the ionomer polymer conformation, are also playing a role in the ion transport through the catalyst layer.

## Summary

**Figure 4:**
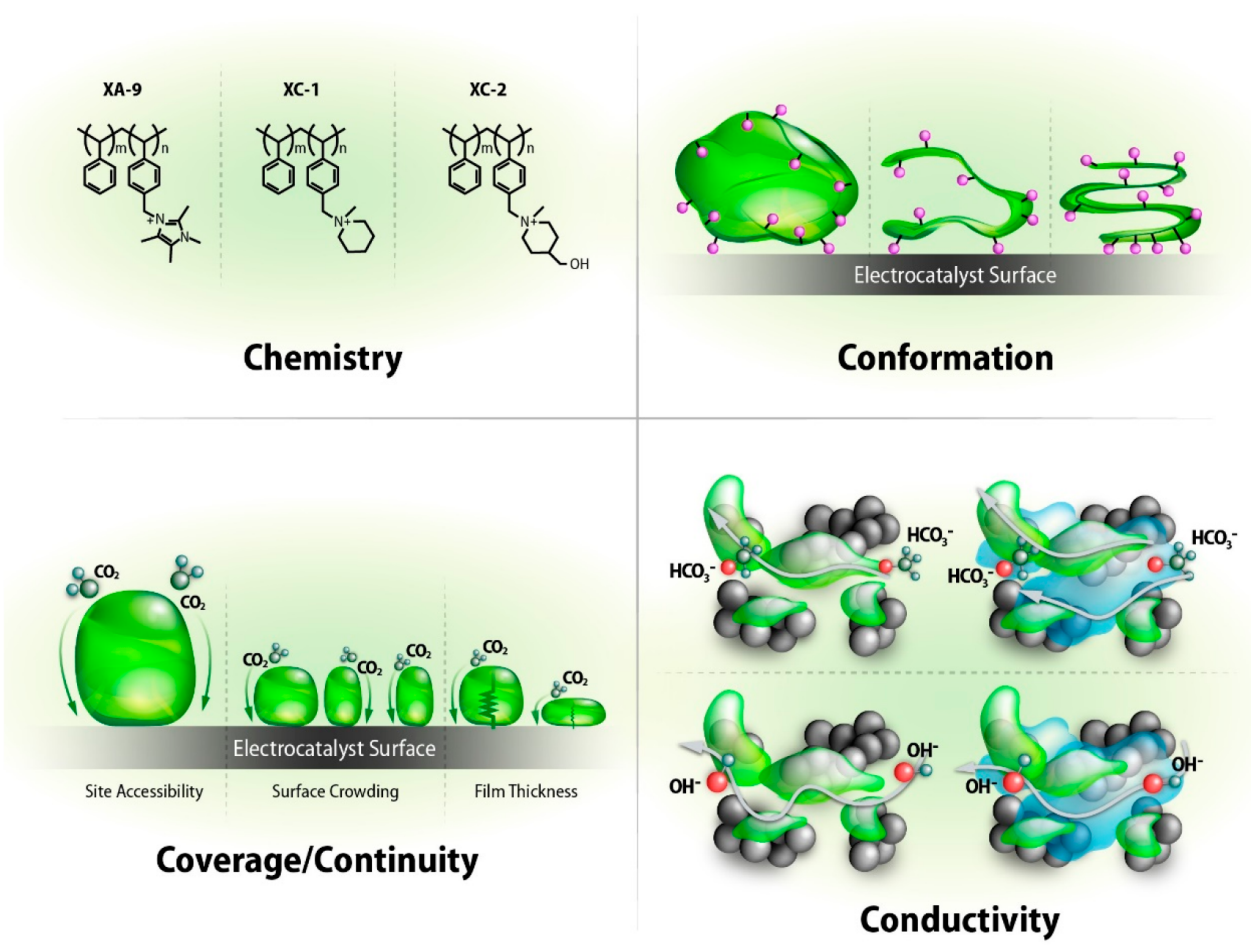
Schematics depicting various ionomer properties (chemistry, confirmation, coverage/continuity, and conductivity) that can impact electrode performance.

In summary, Figure 4 describes the various impacts that ionomers can have on the electrochemical properties of the electrode. The chemistry of the ionomer can affect the hydrophobicity of the catalyst layer, ion conductivity, and reactant gas permeability.^35^ The conformation of the ionomer can also be influenced by the catalyst ink^36,37^ and deposition^22^ method, netting changes in competitive adsorption processes and local gas transport. Significantly thick ionomer films can result in underutilized catalyst sites, where reactant gases are forced around areas with low permeability. Furthermore, changes in ionomer coverage on electrocatalyst sites can influence ionic accessibility to the reaction site, while variations in film thickness influence non-Fickian diffusion resistance.^38^ The breadth of electrode level morphological changes that can occur as ionomer chemistry/properties are modified makes it difficult to elucidate and isolate the impact of said modification. Only through close examination of device level electrochemical properties combined with performance and selectivity assessments can one truly unravel the benefit of the proposed material solutions.

This work demonstrated that, beyond ionomer properties, electrode-level electrochemical phenomena affect CO/H_2_ selectivity at low CO_2_ concentrations. We have identified two important electrode-level parameters that we attribute to differences in ionomer chemistry and/or integration: capacitance increase as a function of RH (related to pore size and/or hydrophobicity) and the resistance of the ion transport (*R*_CL,HCO_3_^–^_) through the catalyst layer. The RH of the cathode feed gas can be an important factor in increasing catalyst utilization, but only if the electrode can distribute water throughout the catalyst layer, which could be influenced by pore size, pore volume, or ionomer hydrophobicity. In scenarios of poor catalyst utilization (low ion transport), electrodes will not have simultaneous access to both gas phase CO_2_ and ions, with the HER favored under these conditions. Ion conduction through the catalyst layer is important for maximum catalyst utilization. This utilization will be especially important in mass transport limited regimes, such as at low CO_2_ concentrations. In this study we find that these electrode level properties influenced by the ionomer affect the performance of the electrode toward conversion of CO_2_ to CO at low concentrations of CO_2_. Figure 4 depicts different ionomer properties, such as ionomer chemistry and conformation, potentially leading to the electrode property level observations observed in this study.

The promising results here may also be due to the high loadings of Ag (∼3 mg/cm^2^), which has been implicated in lowering the neutralized CO_2_ in the catalyst layer due to the higher CO_2_/OH^–^ ratio.^7^ It is clear that every CO_2_ system will have different requirements for optimal performance and we have identified factors that will affect CO_2_ to CO in a zero gap system with a dilute anolyte and a high catalyst loading. Future work will focus on understanding the kinetics of the CO_2_R and HER on Ag electrodes to maximize performance under low reactant access conditions.

## Supporting Information Available

Detailed experimental methods, scanning electron microscopy images and electrochemical impedance spectra (PDF)
