# Supplementary material for: Understanding Limitations in Electrochemical Conversion to CO at Low CO2 Concentrations
Source: ACS Energy Lett. 2024 Jun 24;9(7):3433–9. doi: 10.1021/acsenergylett.4c01224 (PMC11249775; doi:10.1021/acsenergylett.4c01224)
Supplement: Supplementary file 1 — nz4c01224_si_001.pdf [file nz4c01224_si_001.pdf]

Supporting Information for

Understanding Limitations in Electrochemical Conversion to CO at Low CO_2 Concentrations

Danielle A. Henckel[a], Prantik Saha[a], Sunil Rajana[b], Carlos Baez-Cotto[a], Audrey K. Taylor[a], ,

Zengcai Liu[b], Michael G. Resch[a], Richard I. Masel[b] and K. C. Neyerlin[a]*

[a]National Renewable Energy Laboratory, 15013 Denver W Parkway, Golden, CO, 80401, United States

[b]Dioxide Materials, 1100 Holland Dr. Boca Raton, FL, 33487, United States

*Corresponding author, Kenneth.neyerlin@nrel.gov

I. Electrode fabrication

Ag electrodes.

We utilized three different ionomer polymer structures in our Ag cathodes for the results shown here. These are similar in the polystyrene vinyl benzyl backbone with different headgroups- tetramethyl imidazolium (XA-9), 1-methyl piperidinium (XC-2), and 1-methyl, 4-hydroxymethyl piperidinium (XC-1). The cathode ink was created by first mixing 80 mg of solid XA-9 or 250 mg from a 33 wt% solution of XC-1 and XC-2 in DMSO to 5.7-5.8g of 1-methoxy-2-propanol (final ink mass is 10 g). The catalyst, carbon and ionomer ratios were reproduced from published work.[1] Once mixed, 80 mg of XC-72 carbon was added and sonicated until mostly incorporated. To this mixture, 4 g of Ag nanoparticles (U.S. Research Nanomaterials, 20 nm) were added. This formulated recipe contained 40 wt. % catalyst, an ionomer-to-carbon weight ratio of 1, and an ionomer-to-catalyst ratio of 0.02. This ink mixture was then ball-milled with 5 mm zirconia beads on a Thermo Scientific digital bottle roller at 80 rpm or a U.S. Stoneware jar mill roller at 20 speed units (~60 rpm) for 20-26 hours. This ink was then rod-coated onto AvCarb 5130GDS (Fuel Cell Store) by a Qualtech automatic film applicator (QPI-AFA6800). A 5"x8" piece of gas diffusion media was taped to the film applicator, and the hot plate was set to 65°C. Approximately 3 mL of ink was deposited on the gas diffusion media. This ink was drawn down the gas diffusion media with a ½" x 16" wire wound Mayer rod (RD Specialties- 12 mil diameter). These coated gas diffusion electrodes (GDE) were then placed in an 80°C oven for at least 10 min to dry. The 25 cm^2 cathodes were cut with a die cutter. The ionomer within the cathodes was ion exchanged by placing the GDE catalyst side down in 1 M KOH for at least one hour and then rinsed with water and allowed to dry. The average Ag loading was 3.4 mg/cm^2, which was calculated through XRF

IrO_2 electrodes.

The anode ink was made by weighing 1 g of IrO_2 (Premion, 99.98%) and wetting it completely with 1 mL of water first as a safety precaution. Next, 50 mL of IPA and 1 mL of Aquivion 5 wt% were added to this mixture. This ink was horn sonicated for 2x10s and bath sonicated for 1 hour with ice to prevent the ink from over-heating. This mixture was deposited onto 4 pieces of Sigracet 39BB using a Sonotek ExactaCoat with Accumist spray nozzle and an

ink flow rate of 1.0 mL/min onto a hotplate set at 80°C. The average IrO_2 loading was 2.6 mg/cm^2.

Pt/C electrodes.

The catalyst ink was prepared by first combining Pt/HSC (Tanaka Kikinzoku Kyogo (TEC10E50E, 46.7 wt% Pt)) with ultra-pure water (18.2 MΩ) and then adding Nafion D2020, and nPA (Omnisolv® grade) into a 120 mL vial. As a safety precaution, the D2020 and nPA were added last to prevent a Pt–catalyzed combustion of this alcohol. This formulated recipe contained 5 wt. % catalyst, an ionomer-to-carbon weight ratio of 0.20, and a water-to-alcohol weight ratio of 1.5 (60 wt% H_2O). 60 g of ink were dispersed using the Ultra-Turrax® high-shear rotor-stator with (dispersing element 18G) at 10,000 rpm for 30 min. The ink jar was then placed on a Fisherbrand™ Digital Bottle Roller at 80 rpm for 1 h to remove any bubbles. Pt/HSC inks were coated at 22 °C utilizing a ½" x 16" wire wound lab rod (RD Specialties–mil diameter = 30) on a Qualtech automatic film applicator (QPI-AFA6800). 500 µL of catalyst ink deposited on a Freudenberg H23C8 carbon gas diffusion media (Fuel Cell Store) were rod coated at an average speed of 55 mm/s. These electrodes were transferred to an oven and dried at 80 °C. Electrodes were cut to 5 x 5 cm. The average Pt loading is 0.2 mg/cm^2.

II. Cell Assembly

The cell area is 25 cm^2. The anode and cathode flow fields have a triple serpentine pattern and are made of stainless steel and titanium, respectively. The anode and cathodes were surrounded by PTFE gaskets that allowed 20% compression on both electrodes (thickness calculated based on 6% compression of the PTFE and the measured GDE thickness). The Sustainion membranes (Sustainion X37-50 Grade RT) were soaked in 1 M KOH for at least 24 hours before use and rinsed with water before assembly. The cell was tightened first to 20 in-lbs and then to 40 in-lbs. The cell was run at 45°C with 10 mM $KHCO_3$ as the anolyte recirculating at 40 mL/min. The CO_2 flow rate was kept at 2 SLPM, and N_2 was the make-up gas.

III. Cell Testing and Product Analysis

Upon connection to the CO_2 test stand, the cell underwent a break-in procedure that consisted of a 3.0 V hold for 10 minutes. After this hold, the cell was switched immediately to a galvanostatic run at 200 mA/cm^2 (using Gamry sequence wizard). Gas products were analyzed from an Agilent 990 Micro Gas Chromatograph. CO/H_2 selectivity is defined as the molar ratios of $[CO]/([CO]+[H_2])$ in the outlet gas stream. Our conditions of 10% CO_2 at 2 SLPM is a

stoichiometry of 5.2, and 20% at 2 SLPM is a stoichiometry of 10.5 for the CO_2 to CO reaction at 200 mA/cm^2. In this configuration, the minimum stoichiometry is likely 2 or 3; one CO_2 is converted to CO, and one CO_2 molecule in the form of CO_3^- (or two molecules of HCO_3^-) cross the anion exchange membrane toward the anode.[2]

IV. Electrochemical Impedance Spectroscopy

The set-up was similar to that previously published.[3] A Pt/C anode was used as both the reference hydrogen electrode and the counter electrode. Two membranes (Sustainion X37-50 Grade RT) were used for the cathode and anode sides, with a 2 mm electrolyte (polyetherimide) channel between the membranes. 0.2 SLPM of hydrogen flowed at the anode, and 0.2 SLPM of nitrogen flowed at the cathode. A 10 mM $KHCO_3$ electrolyte solution flowed between the membranes at 20 mL/min. The EIS was run in potentiostatic mode at 0.85 V vs RHE (corresponding to the double layer capacitance region), 20 mV rms AC Voltage with an initial frequency of 300,000 Hz and a final frequency of 0.1 Hz.

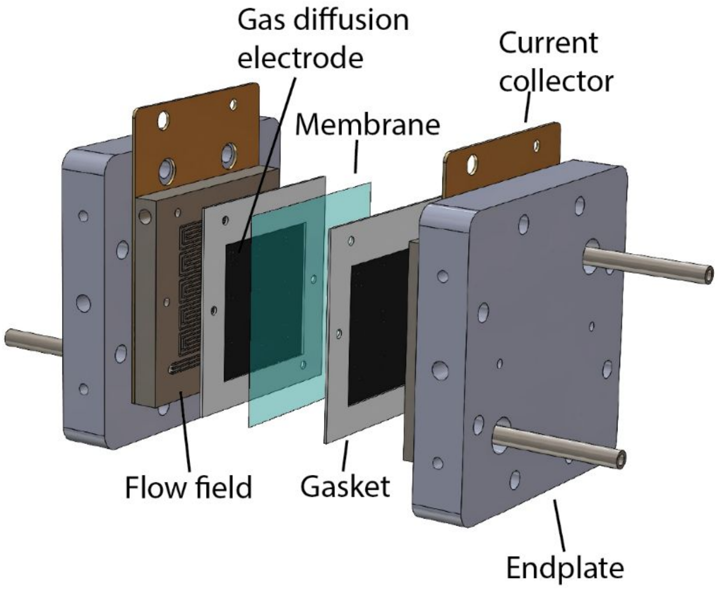

Figure S1. Schematic of the 25 cm^2 cell hardware with labeled components

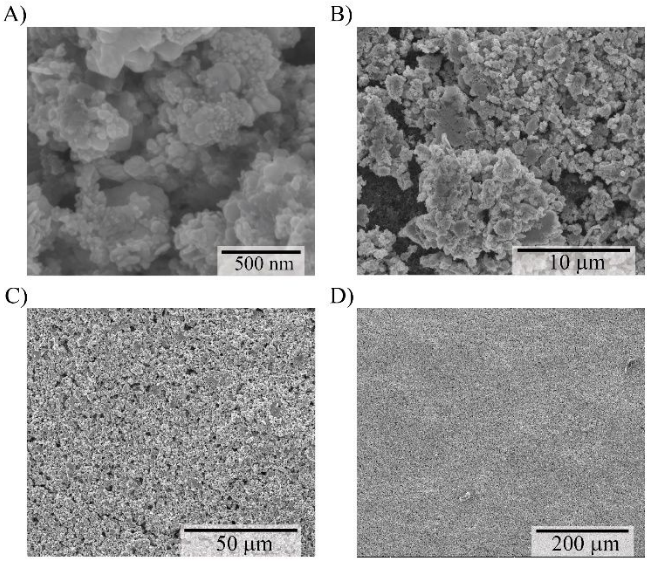

Figure S2. Top-down SEM image of Ag XA-9 electrode

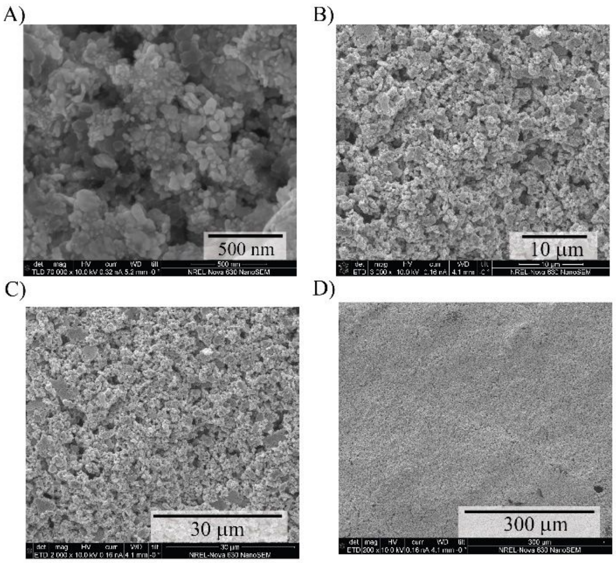

Figure S3. Top-down SEM image of Ag XC-1 electrode

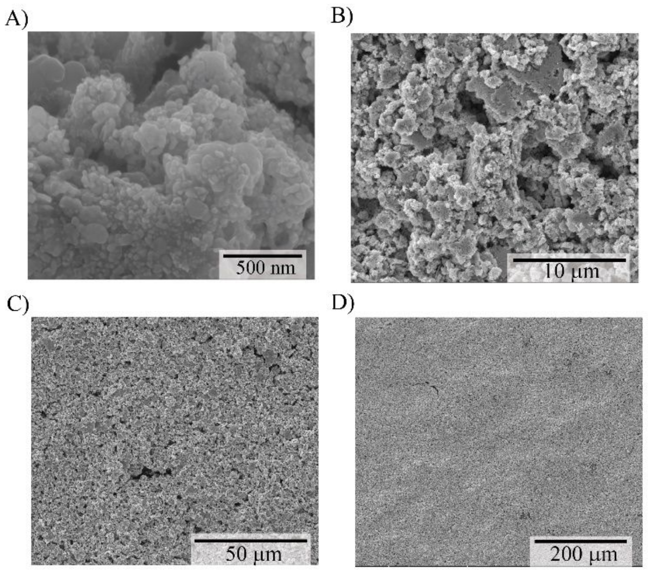

Figure S4. Top-down SEM image of Ag XC-2 electrode

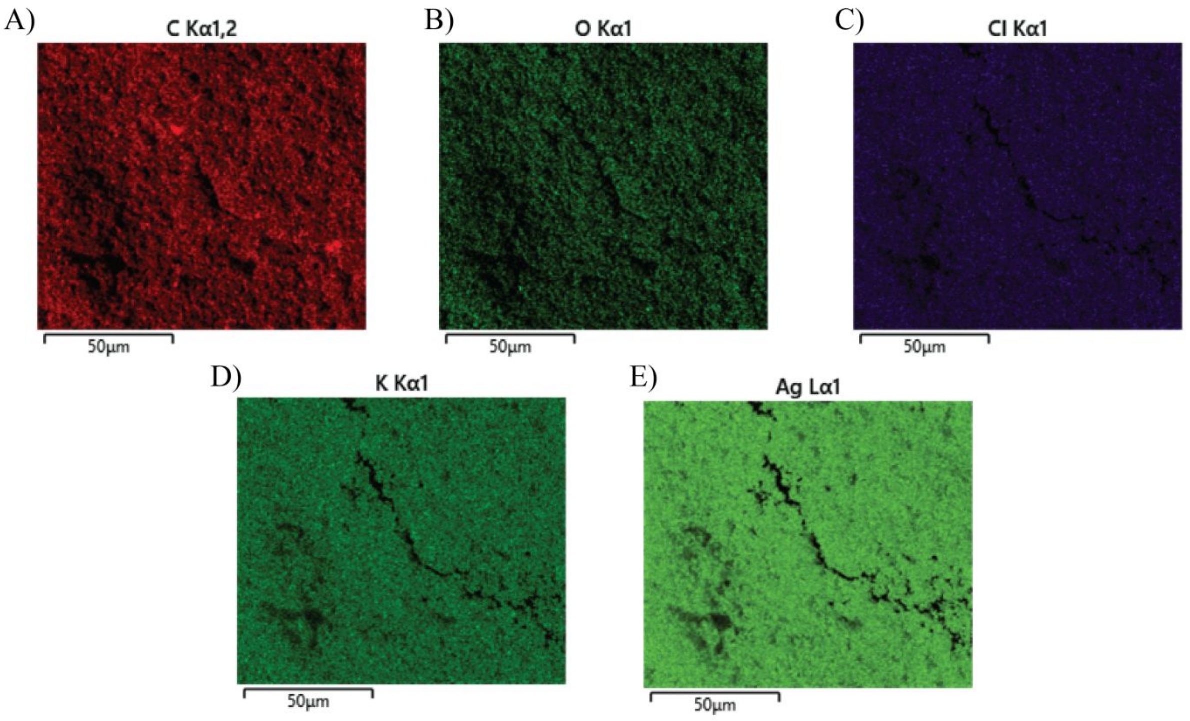

Figure S5. EDS map of Ag XA-9 electrode showing A) C, B) O, C) Cl, D) K and E) Ag

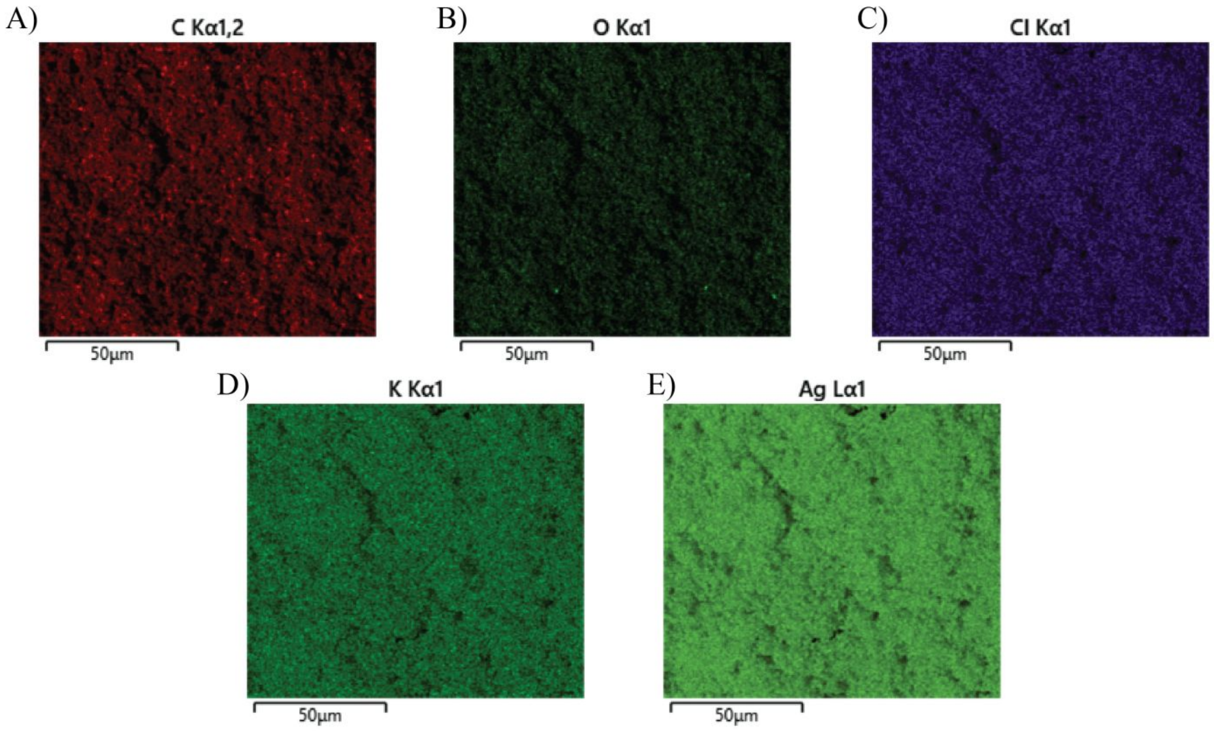

Figure S6. EDS map of Ag XC-1 electrode showing A) C, B) O, C) Cl, D) K and E) Ag

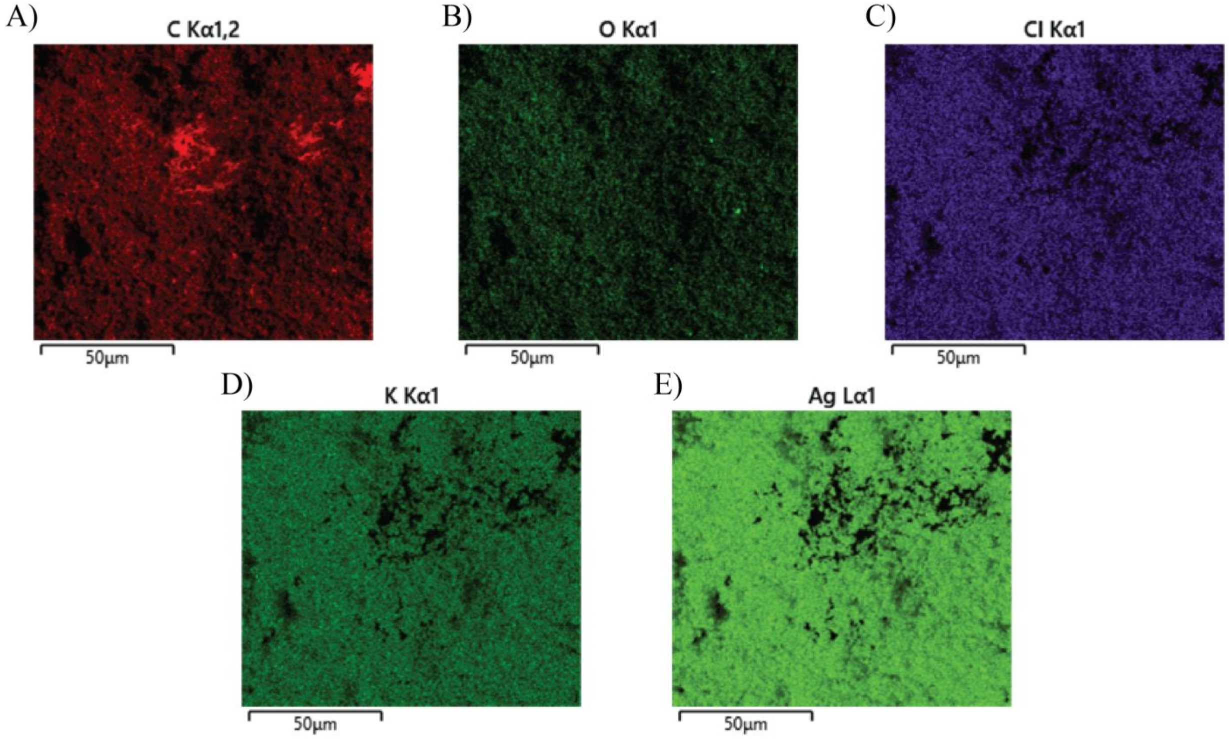

Figure S7. EDS map of Ag XC-2 electrode showing A) C, B) O, C) Cl, D) K and E) A

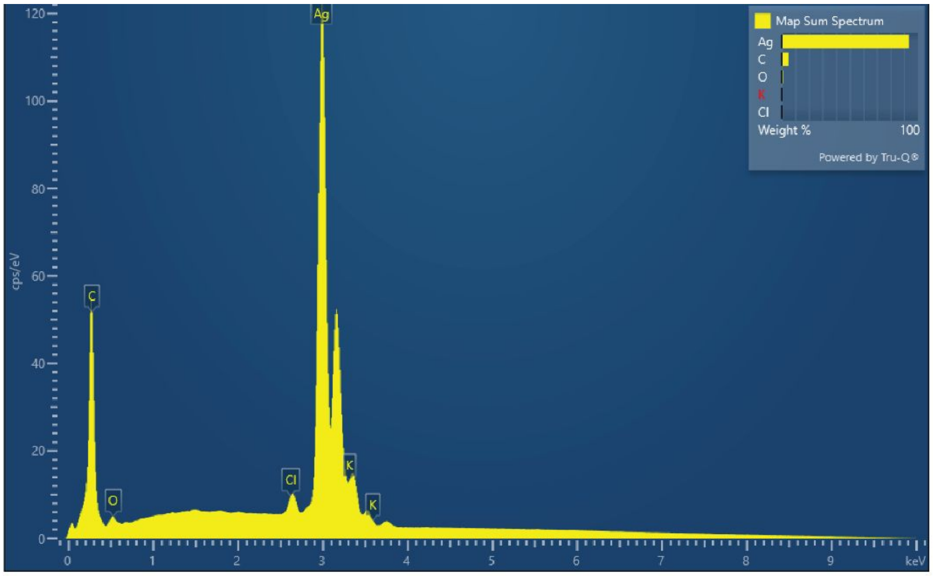

Figure S8. Energy (keV) versus normalized intensity for Ag XA-9 electrode

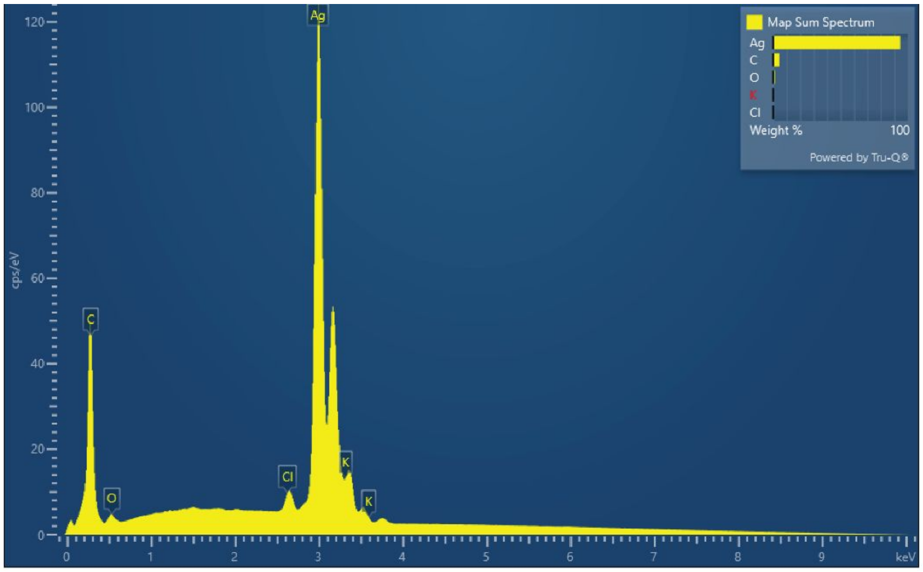

Figure S9. Energy (keV) versus normalized intensity for Ag XC-1 electrode

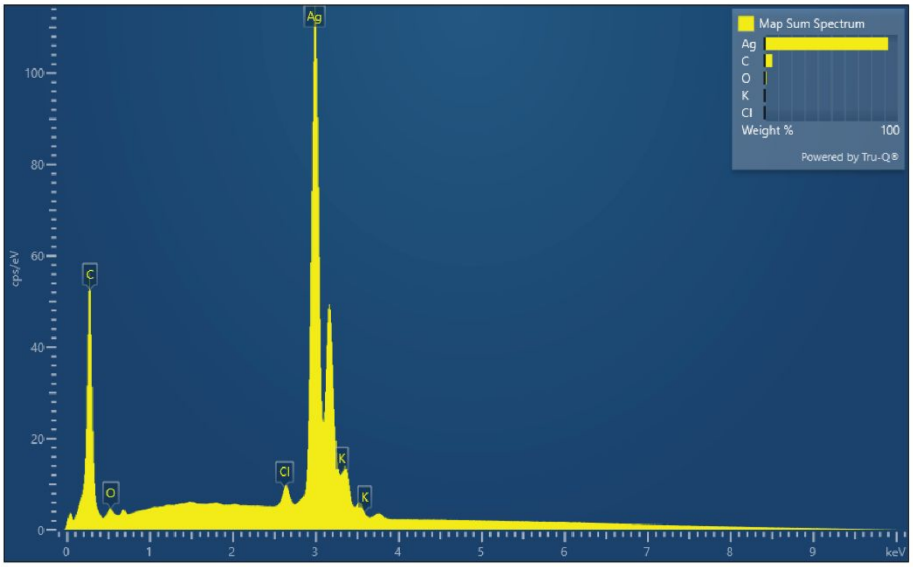

Figure S10. Energy (keV) versus normalized intensity for Ag XC-2 electrode

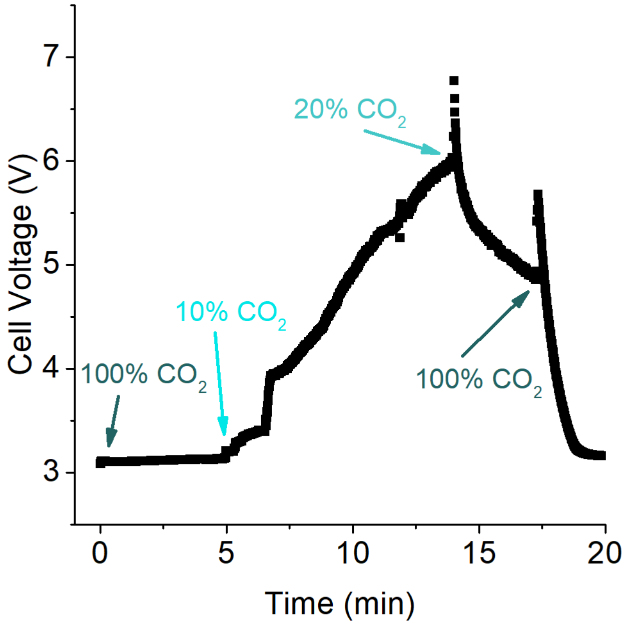

Figure S11. Cell voltage versus time at 200 mA/cm^2 for XC-1 incorporated electrode at various CO_2 concentrations, 50% RH, 2 SLPM total gas flow

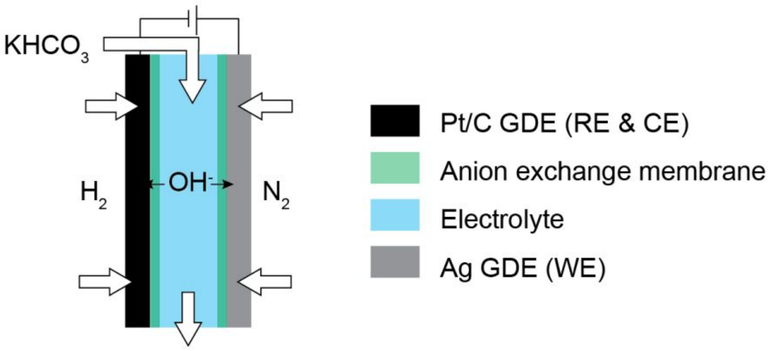

Figure S12. Schematic of the cell for electrochemical impedance spectroscopy (EIS) investigations of the Ag electrodes

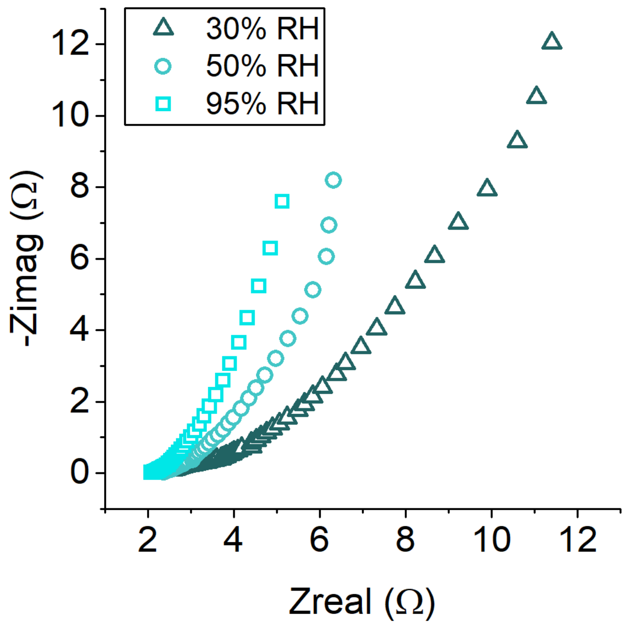

Figure S13. EIS spectra for XA-9 under relative humidities of 30, 50, and 95% (see Experimental and Figure S12 for details)

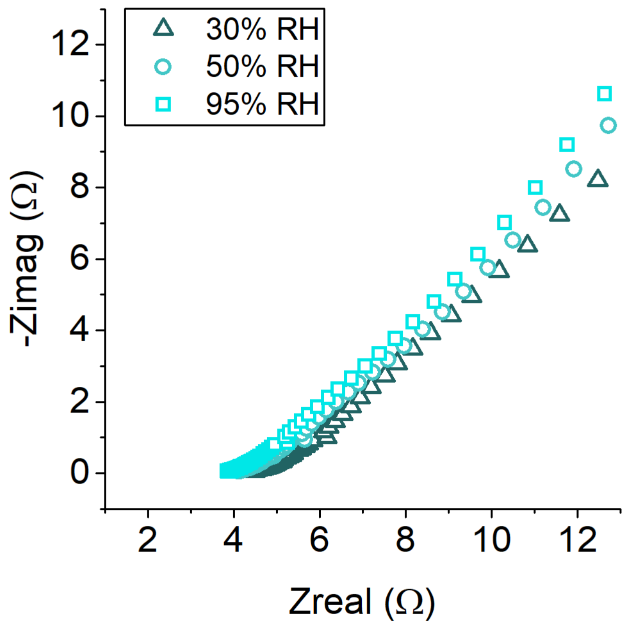

Figure S14. EIS spectra for XC-1 under relative humidities of 30, 50, and 95% (see Experimental and Figure S12 for details)

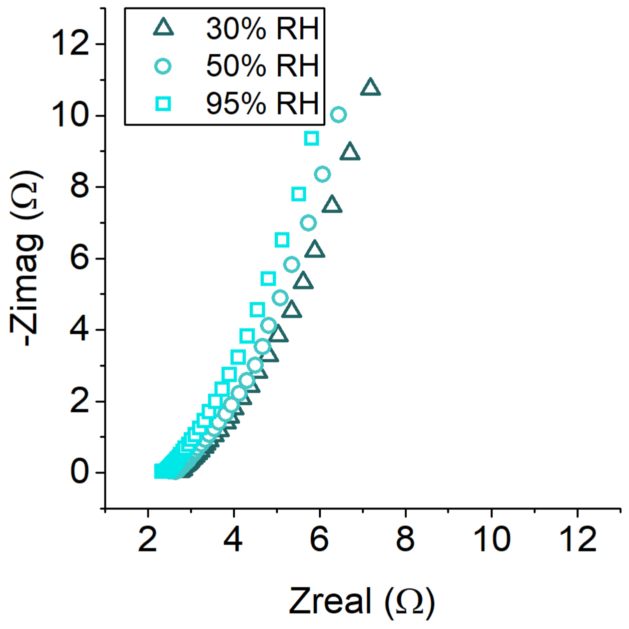

Figure S15. EIS spectra for XC-2 under relative humidities of 30, 50, and 95% (see Experimental and Figure S12 for details)

Table S1. Measured and theoretical ion exchange capacity for the polymer studied.

Ionomer	Measured IEC (mM/g)	Theoretical IEC (mM/g)
XA-9	0.944	2.162
XC-1	1.393	2.139
XC-2	1.394	2.28
